# Supplementary material for: An image-computable model for the stimulus selectivity of gamma oscillations
Source: eLife. 2019 Nov 8;8:e47035. doi: 10.7554/eLife.47035 (PMC6839904; doi:10.7554/eLife.47035)
Supplement: Transparent reporting form [file elife-47035-transrepform.pdf]

eLIFE
1st Floor
24 Hills Road
Cambridge CB2 1JP, UK
P 01223 855340
W elifesciences.org
T @elife

eLife's transparent reporting form

We encourage authors to provide detailed information *within their submission* to facilitate the interpretation and replication of experiments. Authors can upload supporting documentation to indicate the use of appropriate reporting guidelines for health-related research (see EQUATOR Network), life science research (see the BioSharing Information Resource), or the ARRIVE guidelines for reporting work involving animal research. Where applicable, authors should refer to any relevant reporting standards documents in this form.

If you have any questions, please consult our Journal Policies and/or contact us: editorial@elifesciences.org.

Sample-size estimation

- You should state whether an appropriate sample size was computed when the study was being designed
- You should state the statistical method of sample size computation and any required assumptions
- If no explicit power analysis was used, you should describe how you decided what sample (replicate) size (number) to use

Please outline where this information can be found within the submission (e.g., sections or figure legends), or explain why this information doesn't apply to your submission:

Our study consisted of 3 ECoG subjects with 15 electrodes. Analyses were done at the individual electrode level. The data quality was very high, with no concerns about issues of statistical significance. Rather, our interest was in fitting models and interpreting the parameters. Our confidence in the data comes from three sources:

1) The responses per individual stimulus and individual electrode have a very high signal to noise ratio (SNR) of about ~5 for broadband and ~4 for gamma, where signal is the mean and noise is half of the 68% confidence interval from bootstrapping across repeated trials from the same stimulus. *See Figure 3 and 6 and Supplementary Figures*

2) Consistency across electrodes and subjects. The broadband and gamma responses per individual stimulus were consistent across electrodes. Across subjects, the SNR is ~4, where the signal is the mean across electrodes and the noise is half of the 68% confidence interval after bootstrapping across electrodes. *See Figure 2.*

3) Variance explained by model fit. The cross-validated variance explained by the models was extremely high in each individual electrode (ranging from 70% to 89% for broadband and ranging from 38% to 91% for gamma), far beyond the criterion for statistical significance and significantly higher in comparison with other models. *See Figure 3 and 6 and the figure supplements.*

Replicates

- You should report how often each experiment was performed
- You should include a definition of biological versus technical replication
- The data obtained should be provided and sufficient information should be provided to indicate the number of independent biological and/or technical replicates
- If you encountered any outliers, you should describe how these were handled

- Criteria for exclusion/inclusion of data should be clearly stated
- High-throughput sequence data should be uploaded before submission, with a private link for reviewers provided (these are available from both GEO and ArrayExpress)

Please outline where this information can be found within the submission (e.g., sections or figure legends), or explain why this information doesn't apply to your submission:

Our ECoG results are replicated in 3 human subjects across 15 electrodes in total (line number 95-96). Each visual stimulus was repeated 9-15 times (Methods). ECoG electrodes are placed based on a medical indication and not for research reasons. Electrodes over primary visual cortex are rare and to ensure reproducibility, we waited 5 years to collect data on 3 subjects with electrodes over primary visual cortex, but all of whom who had the source of epilepsy far from this region.

Electrode inclusion criteria are reported in the Methods Section "ECoG procedure" and there were 2 sets of criteria:
1) For inclusion in common average rereferencing: *Electrodes that showed large artifacts or showed epileptic activity, as determined by the patient's neurologist were excluded, resulting in 116/107/54 electrodes with a clean signal.*

2) For inclusion in model analysis (Methods section, ECoG procedure): *In further analyses, we only included electrodes that were located on visual areas V1, V2 or V3 with population receptive fields that were within the stimulus (~10 degrees from the fovea).*

There were no outliers or further exclusions.

Statistical reporting

- Statistical analysis methods should be described and justified
- Raw data should be presented in figures whenever informative to do so (typically when N per group is less than 10)
- For each experiment, you should identify the statistical tests used, exact values of N, definitions of center, methods of multiple test correction, and dispersion and precision measures (e.g., mean, median, SD, SEM, confidence intervals; and, for the major substantive results, a measure of effect size (e.g., Pearson's r, Cohen's d)
- Report exact p-values wherever possible alongside the summary statistics and 95% confidence intervals. These should be reported for all key questions and not only when the p-value is less than 0.05.

Please outline where this information can be found within the submission (e.g., sections or figure legends), or explain why this information doesn't apply to your submission:

Our analyses are based on model fitting. We report the variance explained (formula given in section about Model Accuracy) in the data by models, which had a mean of 75% (range from 38-91% across electrodes) explained for gamma and a mean of 80% (range from 71% to 89% across electrodes) explained by broadband. The explained variance is provided for every electrode in Figure 3-6 supplement 1-2 and in Figure 7.

The responses to each individual stimulus are also shown for all electrodes in main Figure 2, 3 and 6, figure 3 - supplements 1-2 and figure 6 - supplements 1-2. Responses to each stimulus are plotted as the mean and 68% confidence interval from bootstrapping. P-values were calculated based on bootstrapping (Methods section on Significance Testing).

(For large datasets, or papers with a very large number of statistical tests, you may upload a single table file with tests, Ns, etc., with reference to sections in the manuscript.)

Group allocation

- Indicate how samples were allocated into experimental groups (in the case of clinical studies, please specify allocation to treatment method); if randomization was used, please also state if restricted randomization was applied
- Indicate if masking was used during group allocation, data collection and/or data analysis

Please outline where this information can be found within the submission (e.g., sections or figure legends), or explain why this information doesn't apply to your submission:

There were no groups.

Additional data files ("source data")

- We encourage you to upload relevant additional data files, such as numerical data that are represented as a graph in a figure, or as a summary table
- Where provided, these should be in the most useful format, and they can be uploaded as "Source data" files linked to a main figure or table
- Include model definition files including the full list of parameters used
- Include code used for data analysis (e.g., R, MatLab)
- Avoid stating that data files are "available upon request"

Please indicate the figures or tables for which source data files have been provided:

All data used in this manuscript will be made available in the standardized, open source, data structure for intracranial EEG data: iEEG-BIDS (Gorgolewski et al., 2016 Scientific Data and Holdgraf et al., PsyArXiv). These data and the code to reproduce each figure are provided on the OSF platform: https://osf.io/eqjxb/?view_only=a0244a0e352c42b98f5e19b3374c84e8

As in previous work, Matlab code is provided to analyze the data reproduce each figure with a specific script: /make_figures/makeFigX.m
